# Disruption of *Slc52a3* gene causes neonatal lethality with riboflavin deficiency in mice

**DOI:** 10.1038/srep27557

**Published:** 2016-06-08

**Authors:** Hiroki Yoshimatsu, Atsushi Yonezawa, Kaori Yamanishi, Yoshiaki Yao, Kumiko Sugano, Shunsaku Nakagawa, Satoshi Imai, Tomohiro Omura, Takayuki Nakagawa, Ikuko Yano, Satohiro Masuda, Ken-ichi Inui, Kazuo Matsubara

**Affiliations:** 1 Department of Clinical Pharmacology and Therapeutics, Kyoto University Hospital, Sakyo-ku, Kyoto 606-8507, Japan

## Abstract

Homeostasis of riboflavin should be maintained by transporters. Previous *in vitro* studies have elucidated basic information about riboflavin transporter RFVT3 encoded by *SLC52A3* gene. However, the contribution of RFVT3 to the maintenance of riboflavin homeostasis and the significance *in vivo* remain unclear. Here, we investigated the physiological role of RFVT3 using *Slc52a3* knockout (*Slc52a3*−/−) mice. Most *Slc52a3*−/− mice died with hyperlipidemia and hypoglycemia within 48 hr after birth. The plasma and tissue riboflavin concentrations in *Slc52a3*−/− mice at postnatal day 0 were dramatically lower than those in wild-type (WT) littermates. *Slc52a3*−/− fetuses showed a lower capacity of placental riboflavin transport compared with WT fetuses. Riboflavin supplement during pregnancy and after birth reduced neonatal death and metabolic disorders. To our knowledge, this is the first report to indicate that Rfvt3 contributes to placental riboflavin transport, and that disruption of *Slc52a3* gene caused neonatal mortality with hyperlipidemia and hypoglycemia owing to riboflavin deficiency.

The water-soluble vitamin B2 (riboflavin) is involved in various metabolic reactions. It is converted to active coenzyme forms, flavin mononucleotide (FMN) and flavin adenine dinucleotide (FAD), which are required as cofactors for redox reactions, including carbohydrate, lipid, and amino acid metabolism. Riboflavin is essential for cellular function; deficiency of riboflavin leads to various clinical abnormalities (e.g. anemia, neurodegeneration and growth retardation)^1^,^2^. The low maternal riboflavin intake contributes to the risk of some disorders in offspring, like congenital heart defects and transverse limb deficiency^2^,^3^,^4^, suggesting the necessity of riboflavin for healthy fetal growth.

Transporters play an important role in riboflavin homeostasis^5^. Until now, three riboflavin transporters have been identified, and designated as the RFVT family (RFVT1-3)^6^,^7^,^8^,^9^. Riboflavin transporter 3 (RFVT3) encoded by the *SLC52A3* gene was identified based on the sequence of bacterial riboflavin transporter impX in 2009^7^. Previous *in vitro* studies have extensively documented basic information about RFVT3^7^,^8^,^9^,^10^,^11^,^12^,^13^. This transporter consists of 469 amino acids with 11 putative membrane-spanning domains, and is strongly expressed in the small intestine, kidney, testis and placenta^7^,^8^. The results of previous studies indicate that RFVT3 can transport riboflavin^8^; however, riboflavin analogs, FMN and FAD, are poor substrates for RFVT3^13^. In addition, recent *in vitro* studies suggest that RFVT3 is functionally involved in intestinal riboflavin absorption in the apical membranes of intestinal epithelial cells^12^,^13^.

Recently, several clinical reports have mentioned that *SLC52A3* mutations have been detected in patients with a neurological disorder (Brown-Vialetto-Van Laere syndrome; BVVLS), wherein fatty acid metabolism is impaired^14^,^15^,^16^,^17^,^18^,^19^,^20^,^21^. BVVLS is an orphan disease as only 92 patients have been documented prior to 2014^22^. In patients with *SLC52A3* mutation, plasma riboflavin levels tend to be lower than those in healthy subjects despite normal dietary intake^16^. In several case reports, the neurological symptoms and abnormal fatty acid metabolism of patients improved after supplementation with riboflavin^14^,^16^,^17^,^18^. Based on these limited clinical reports, disruption of *SLC52A3* might disturb riboflavin homeostasis and trigger BVVLS. However, the contribution of RFVT3 to the maintenance of riboflavin homeostasis and its physiological significance *in vivo* remain unclear.

In this *in vivo* study, we investigated the impact of Rfvt3 on riboflavin homeostasis using *Slc52a3* knockout (*Slc52a3*−/−) mice. *Slc52a3*−/− mice exhibited several phenotypes including riboflavin deficiency and neonatal sudden death. Importantly, their conditions were improved by riboflavin supplementation. We demonstrated for the first time that Rfvt3 contributes to placental riboflavin transport, and disruption of *Slc52a3* caused neonatal lethality with hyperlipidemia and hypoglycemia owing to riboflavin deficiency.

## Results

### Targeted disruption of the *Slc52a3* gene

*Slc52a3*−/− mice in C57BL/6 background were obtained from the Knockout Mouse Project (KOMP) Repository. The mouse *Slc52a3* gene was integrated with the SA-βGeo-pA trapping cassette between exons 1 and 2 (Supplementary Fig. S1). In genomic long-range PCR analysis, the integrated *Slc52a3* allele was detected in the heterozygous mice, indicating that the SA-βGeo-pA trapping cassette was correctly targeted to the *Slc52a3* locus. Heterozygous male and female mice were mated, and the targeted *Slc52a3* allele was detected in these offspring genomic DNA isolated from tail biopsies by PCR analysis. To confirm the disruption of the *Slc52a3* gene, *Slc52a3* mRNA expression was examined. Real-time PCR analysis demonstrated that *Slc52a3* mRNA in the kidney was hardly detected in *Slc52a3*−/− mice, and was significantly lower in *Slc52a3*+/− mice than in WT mice.

### Phenotypic analysis and riboflavin homeostasis in *Slc52a3*−/− mice

**Figure 1:**
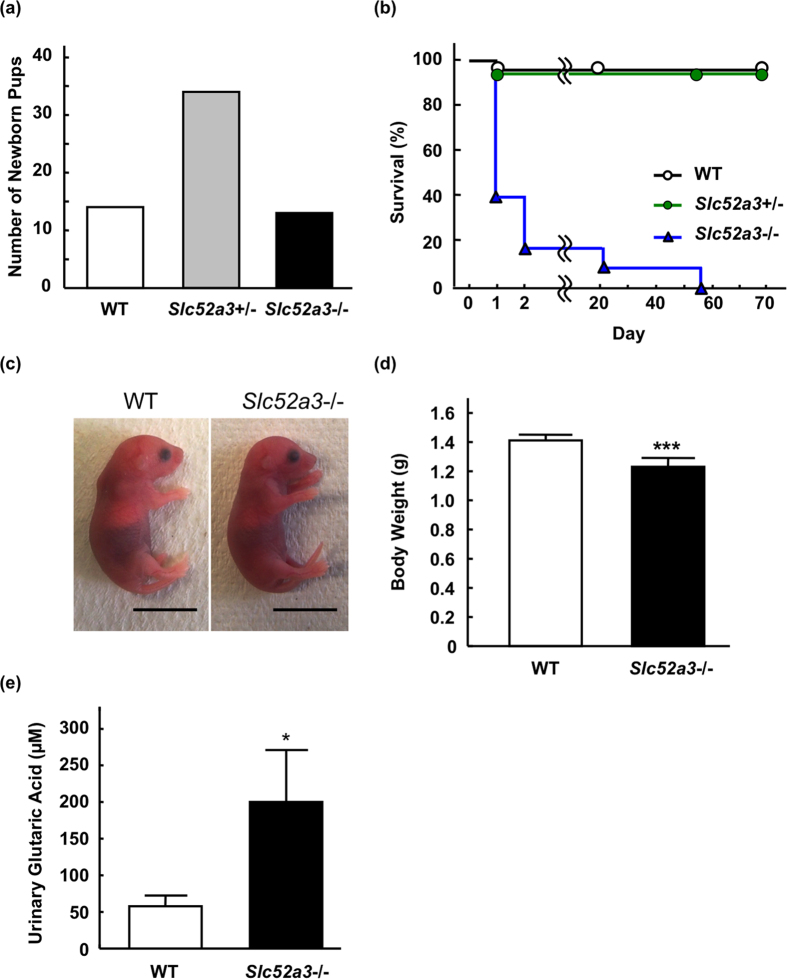
Phenotypic analyses of *Slc52a3*−/− mice. (**a**) Genotypic distribution immediately after birth (n = 63 from 8 litters). (**b**) Kaplan-Meier survival curves for WT (n = 14; open circles, black line), *Slc52a3*+/− (n = 34; closed circles, green line) and *Slc52a3*−/− (n = 13; closed triangles, blue line) littermates. (**c**) Gross appearance of whole body of WT, *Slc52a3*+/− and *Slc52a3*−/− newborn pups at postnatal day 0 (P0). (**d**) Body weight of WT, *Slc52a3*+/− and *Slc52a3*−/− littermates at P0. (**e**) Urinary glutaric acid levels in WT and *Slc52a3*−/− littermates at P0. Each scale bar represents 1 cm length. Each column or point represents the mean ± SD. Values where *P < 0.05, or ***P < 0.001, significantly different from WT.

Genotyping of newborn pups from *Slc52a3*+/− parents (n = 8) at birth revealed no fetal wastage; the mutant allele segregated in Mendelian fashion (*P* = 0.85, Fig. 1a). Most of the newborn *Slc52a3*−/− mice were alive for more than 8 hr, but died within 48 hr after birth. Survival times of *Slc52a3*−/− mice were significantly shorter than those of WT mice (*P* < 0.001, Fig. 1b). Macroscopically, although their appearance was not visibly different from those of WT mice (Fig. 1c), the body weights of *Slc52a3*−/− mice were significantly lower than those of WT mice at P0 (Fig. 1d). Organogenesis appeared to be macroscopically normal (Supplementary Fig. S2). The amount of maternal milk ingestion seemed to be relatively lower in *Slc52a3*−/− pups compared with that in WT pups. The lungs of *Slc52a3*−/− mice floated, indicating that the initial postnatal expansion occurred normally. *Slc52a3*−/− pups as well as WT pups could exercise their arms and legs. Histological analysis of femoral muscle tissues demonstrated that no obvious abnormality was found in *Slc52a3*−/− pups (Supplementary Fig. S3). In addition, apoptosis-inducing factor, mitochondrion-associated 1 (AIFM1) expression levels were also similar in femoral muscles in WT and *Slc52a3*−/− pups. Urinary glutaric acid, a marker for fatty acid metabolism disorder, was significantly higher in *Slc52a3*−/− mice (*P* < 0.05, Fig. 1e).

**Figure 2:**
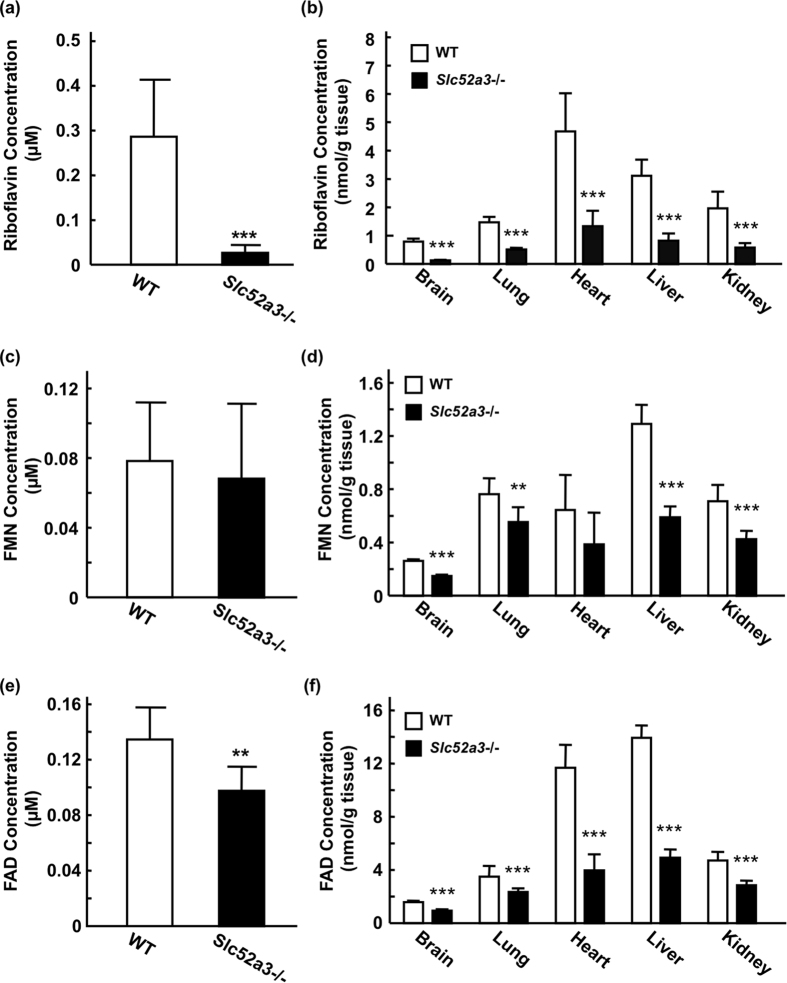
Riboflavin, FMN, and FAD levels in plasma and tissues from WT and *Slc52a3*−/− mice. Plasma and tissues were obtained from WT (n = 14) and *Slc52a3*−/− (n = 13) littermates at postnatal day 0 (P0) immediately after birth. Plasma concentrations of riboflavin (**a**), FMN (**c**) and FAD (**e**) were measured by HPLC. Tissue levels of riboflavin (**b**), FMN (**d**) and FAD (**f**) were also determined. Each column represents the mean ± SD. Values where **P < 0.01, ***P < 0.001, significantly different from WT.

Plasma riboflavin levels in *Slc52a3*−/− mice at P0 were dramatically lower than those in WT mice (Fig. 2a). Additionally, plasma concentrations of FAD, but not FMN, were also decreased in *Slc52a3*−/− mice compared with WT mice (Fig. 2c,e). In almost all tissues examined, the disruption of *Slc52a3* led to not only significantly lower riboflavin but also FMN and FAD levels (Fig. 2b,d,f).

### Role of Rfvt3 in the placental transport of riboflavin

**Figure 3:**
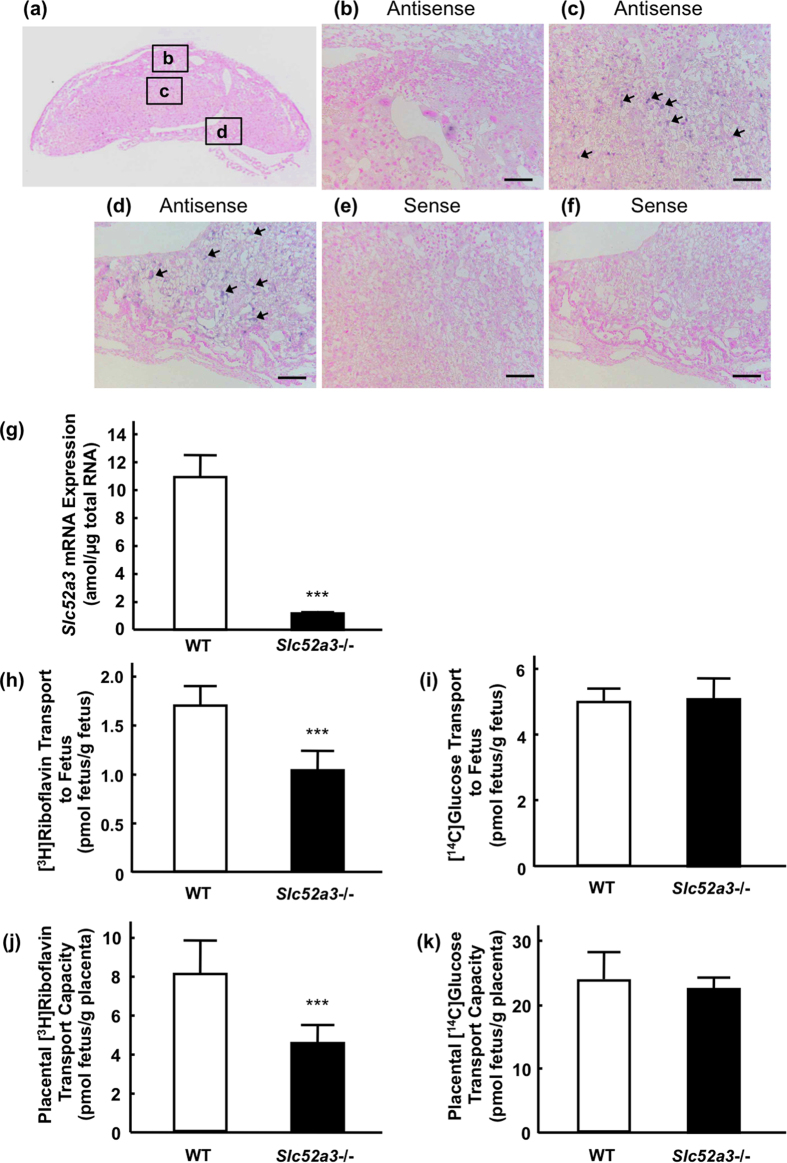
Involvement of Rfvt3 in the placental riboflavin transport. (**a**) mRNA expression of *Slc52a3* was examined by *in situ* hybridization. The insets indicate the magnification region. (**b–d**) Higher magnification images corresponding the solid flames in (**a**): deciduum (**b**), junctional zone (**c**) and labyrinth zone (**d**). The blue signals were positive staining for *Slc52a3* (arrows). Hybridization with a sense probe for *Slc52a3* (negative control) in the junctional zone (**e**) and labyrinth zone (**f**). Scale bar: 100 μm. (**g**) *Slc52a3* mRNA expression in the placentas from WT and *Slc52a3*−/− fetuses derived from *Slc52a3*+/− dams. Total RNA isolated from the placentas from WT and *Slc52a3*−/− fetuses was reverse-transcribed and mRNA level of *Rfvt3* was determined by real-time PCR. Each column represents the mean ± SD (WT, n = 6; *Slc52a3*−/−, n = 6 from 2 litters). (**h,i**) Placental transport of [^3^H]riboflavin in WT and *Slc52a3*−/− fetuses. *Slc52a3*+/− mice were mated, and [^3^H]riboflavin was administered intravenously to pregnant dams at gestation day 16.5. Five minutes after administration, fetuses and placentas were collected. Placental transport of [^3^H]riboflavin was expressed per gram of placenta (**h**) or per gram of fetus (**i**). Placental transport of [^14^C]glucose was also examined (**j,k**). Each column represents the mean ± SD (WT, n = 7; *Slc52a3*−/−, n = 8 from 3 litters). Values where ***P < 0.001 were significantly different from WT.

The mRNA distribution of *Slc52a3* in the mouse placenta was examined by *in situ* hybridization (Fig. 3a). Positive staining for *Slc52a3* was detected in the junctional and labyrinth zones in the placenta (Fig. 3c,d). *Slc52a3* mRNA was not detected in the deciduum (Fig. 3b). Hybridization with a sense-probe for *Slc52a3* showed an absence of nonspecific binding to the junctional and labyrinth zones (Fig. 3e,f). Real-time PCR analysis confirmed the disruption of *Slc52a3* in the placenta of *Slc52a3*−/− fetuses (Fig. 3g). We next investigated the functional involvement of Rfvt3 in placental riboflavin transport. In *Slc52a3*−/− fetuses, the body and placenta weights were significantly lower than in the WT mice (Table 1). Fetal and placental weight ratios did not differ between the WT and *Slc52a3*−/− fetuses. Upon intravenous administration of [^3^H]riboflavin to pregnant dams, *Slc52a3*−/− fetuses exhibited significantly lower [^3^H]riboflavin levels (Fig. 3h). Placental [^3^H]riboflavin transport capacities (adjusted by placental weight) in *Slc52a3*−/− fetuses were also significantly lower than in WT fetuses (Fig. 3i). We confirmed that placental transport of D-[U-^14^C]glucose was not significantly affected by knockout of *Slc52a3* (Fig. 3j,k).

### Riboflavin supplementation in Slc52a3−/− mice

**Figure 4:**
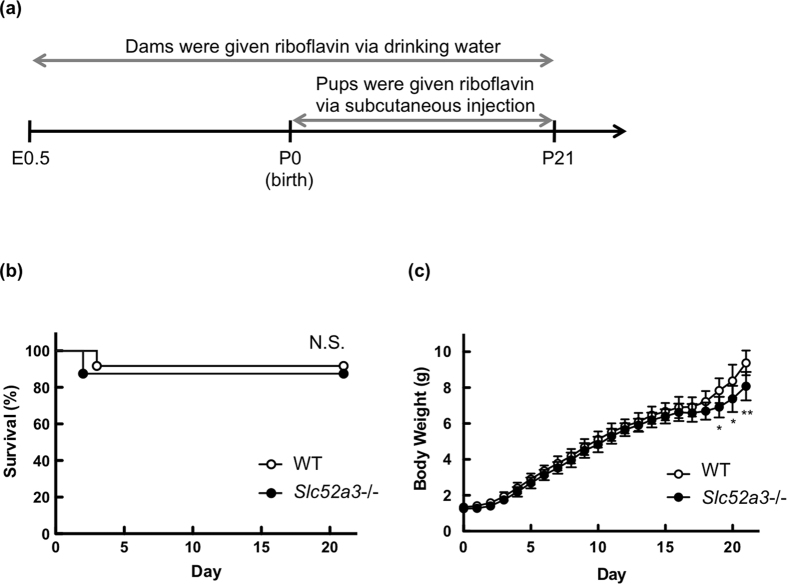
Survival of *Slc52a3*−/− mice with riboflavin supplementation. (**a**) Protocol for the riboflavin supplementation. *Slc52a3*+/− mice were mated, and pregnant dams were supplemented with 50 mg/L of riboflavin in drinking water *ad libitum* from gestational day 0 to 3 weeks postpartum. Newborn pups were also administered 0.75 mg/kg riboflavin subcutaneously once a day until weaning at the postnatal day 21. Kaplan-Meier survival (**b**) and body weights (**c**) for WT and *Slc52a3*−/− mice with riboflavin administration. The body weight values are exressed as the mean ± SD for mice that were alive at each time point (WT, n = 12; *Slc52a3*−/−, n = 8 from 4 litters).

We then examined the effect of riboflavin supplementation on the survival of *Slc52a3*−/− mice (n = 8) compared with WT littermates (n = 12). Briefly, *Slc52a3*+/− mother mice were supplemented with riboflavin (50 mg/L) in drinking water and newborn pups were given a daily subcutaneous injection of 0.75 mg/kg riboflavin until weaning at the P21 (Fig. 4a). Under the riboflavin treatment, the survival of *Slc52a3*−/− pups (n = 7) was comparable to that of WT littermates (n = 11) (Fig. 4b). In terms of body weights, the values in *Slc52a3*−/− mice tended to be lower than those in WT littermates (Fig. 4c).

**Figure 5:**
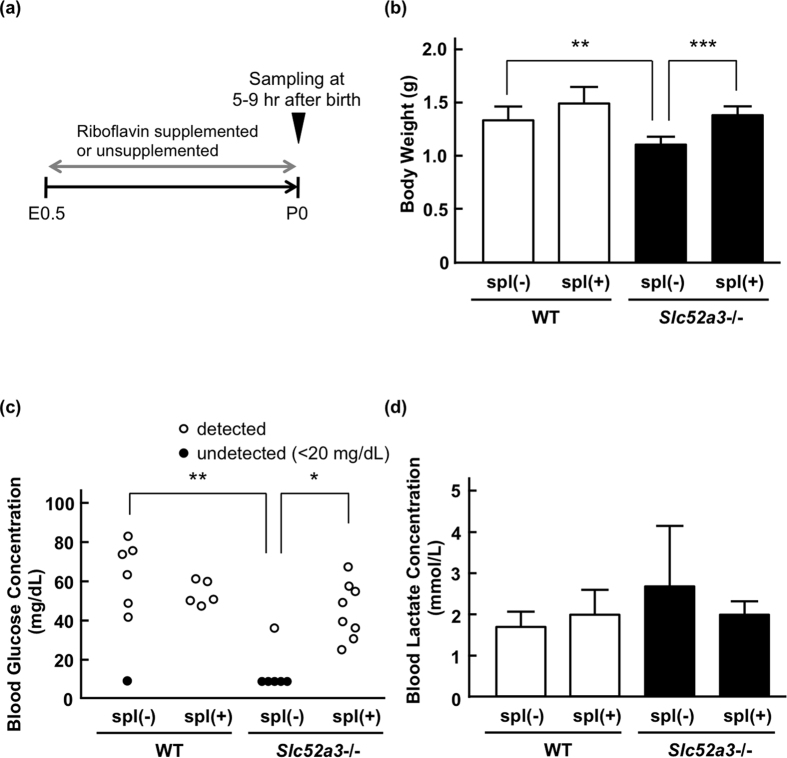
Blood glucose and lactate levels in *Slc52a3*−/− neonatal mice with or without riboflavin supplementation. (**a**) Protocol for the riboflavin supplementation. In the riboflavin-supplemented group (spl (+)), the pregnant dams were given 50 mg/L of riboflavin in drinking water *ad libitum* from gestational day 0. Postnatal day 0 (P0) pups were collected at 5–9 hr after birth. (**b**) Body weights of WT and *Slc52a3*−/− pups. (**c**) Blood glucose levels in WT and *Slc52a3*−/− pups. Closed circles represent undetected data (<20 mg/dL). (**d**) Blood lactate levels in WT and *Slc52a3*−/− pups. Each column represents the mean ± SD (WT spl(−), n = 7; WT spl(+), n = 5; *Slc52a3*−/− spl(−), n = 6; *Slc52a3*−/− spl(+), n = 8). Values where *P < 0.05, **P < 0.01, or ***P < 0.001 indicates significant difference.

We evaluated the plasma acylcarnitine and blood glucose levels in *Slc52a3*−/− pups with or without riboflavin supplementation. *Slc52a3*+/− dams were supplemented with riboflavin during pregnancy, and newborn pups were sacrificed 5–9 hr after birth (Fig. 5a). Plasma riboflavin levels were significantly (*P* < 0.05) increased in *Slc52a3*−/− pups when their dams were given riboflavin via drinking water (Supplementary Table S1). In almost all tissues examined, the concentrations of FMN and FAD were also increased by riboflavin supplementation. Weight loss at birth was significantly improved in supplemented *Slc52a3*−/− pups (Fig. 5b). Analysis of plasma acylcarnitine levels in untreated *Slc52a3*−/− neonatal mice demonstrated elevated levels of several acylcarnitines (C4, C5, and C14) compared with those in WT mice (Table 2). These abnormalities of acylcarnitine in *Slc52a3*−/− mice were significantly improved by riboflavin supplementation (Table 2). Furthermore, in most of *Slc52a3*−/− pups, blood glucose levels were undetectable (<20 mg/dL) (Fig. 5c). Glucose levels in *Slc52a3*−/− pups were significantly increased by riboflavin supplementation (Fig. 5c). To confirm the influence of riboflavin supplementation on blood glucose levels, we conducted an experiment using adult *Slc52a3*−/− mice as shown in Supplementary Fig. S4. At 3-week old (riboflavin treated condition), the blood glucose levels in *Slc52a3*−/− mice were comparable to those in WT mice. However, two weeks after terminating riboflavin supplementation (at 5-week old), the blood glucose levels in *Slc52a3*−/− mice were significantly lower than those in WT mice. As for the blood lactate levels at P0, there was no significant difference between WT and *Slc52a3*−/− pups (Fig. 5d).

**Figure 6:**
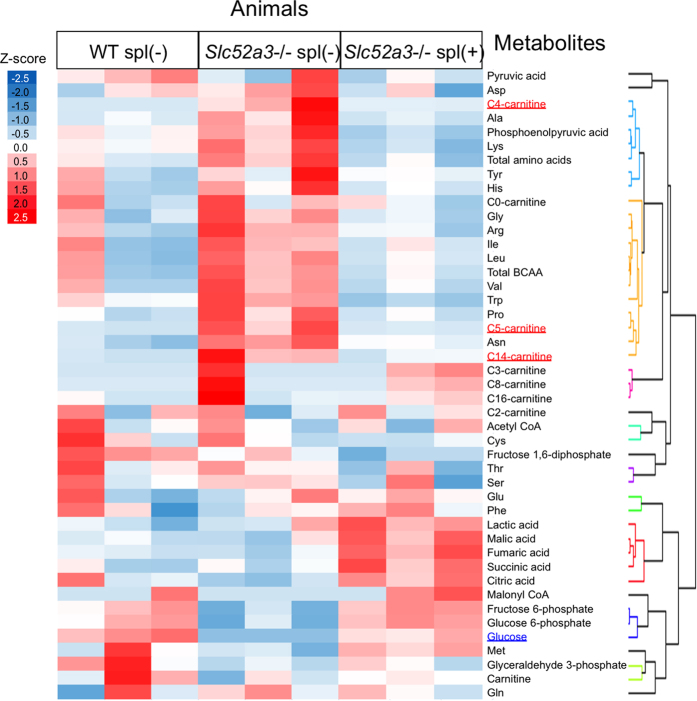
Metabolome analysis in *Slc52a3*−/− neonatal mice. Hierarchical clustering of targeted metabolomics data. Amino acids, metabolites of glycolysis and TCA cycle in liver, plasma acylcarnitine, and blood glucose in WT, Slc52a3−/− pups (Slc52a3−/− spl(−)) and riboflavin-supplemented Slc52a2−/− pups (Slc52a3−/− spl(+)) are listed on the abscissa. The color in the heat map reflects the relative metabolite abundance level according to the z-score.

To identify the biochemical mechanisms underlying the hypoglycemia, targeted quantitative metabolomics, focusing on glycolysis, tricarboxylic acid cycle and amino acid metabolites, was performed using liver samples obtained from WT, *Slc52a3*−/− and riboflavin-supplemented *Slc52a3*−/− pups at P0. The data from the metabolomics analysis and acylcarnitine levels were simultaneously analyzed by hierarchical cluster analysis (Fig. 6). The metabolites of upstream glycolysis (glucose-6-phosphate, and fructose-6-phosphate) were placed in the same classification as glucose (lower in *Slc52a3*−/− than WT pups). Most amino acids, including a gluconeogenic precursor alanine, belong to the same group as C4-acylcarnitine, showing that these levels tended to be higher, but not lower, in *Slc52a3*−/− pups than in WT pups or riboflavin-supplemented *Slc52a3*−/− pups.

## Discussion

In this study, we investigated the physiological role of Rfvt3 *in vivo* using *Slc52a3*−/− mice. Pups from *Slc52a3*+/− parents showed a Mendelian distribution of the genotypes, while *Slc52a3*−/− pups exhibited the severe phenotype of sudden death almost within 48 hr after birth. The appearance of tissues obtained from these pups was macroscopically normal, and lung tissues floated in water. These data suggest that *Slc52a3* null embryos were not lethal *in utero*, and that *Slc52a3*−/− pups seemed not to have died from respiratory compromise or developmental defects in the tissues. In *Slc52a3*−/− newborn pups, plasma and tissue riboflavin concentrations were prominently lower compared with those in WT littermates. As for FMN, tissue level in *Slc52a3*−/− pups was significantly lower than that in WT pups, although the plasma level was comparable. It was reported that FMN and FAD are poor substrates for RFVTs, compared with riboflavin^8^,^13^. FMN and FAD are generated from riboflavin by riboflavin kinase and FAD synthetase in the cells. Therefore, it was suggested that the tissue levels of FMN and FAD in *Slc52a3*−/− pups were lower than those in WT pups, owing to a decrease in the tissue riboflavin concentration. Most importantly, riboflavin level was significantly recovered and neonatal death was apparently reduced in *Slc52a3*−/− pups by riboflavin supplementation. Taken together, Rfvt3 is indispensable for the maintenance of riboflavin homeostasis in pups and for neonatal survival.

It has been suggested that placental riboflavin transport is mediated by transporters. Studies with perfused human placental tissues have identified a saturable riboflavin uptake process^23^,^24^. This active riboflavin transport has also been observed in human trophoblast-derived BeWo cell monolayers^25^. However, the molecular entity of the placental riboflavin transporter and its physiological importance are poorly understood as yet. We have previously reported that RFVT3 is expressed in the human placenta^8^, and the present study demonstrated the significant role of Rfvt3 in the placental riboflavin transport *in vivo*. *In situ* hybridization revealed that Rfvt3 was expressed in the labyrinth zone, which is the feto-maternal exchange site in the placenta. Furthermore, the placental riboflavin transport capacity in *Slc52a3*−/− fetuses was significantly lower than that in WT fetuses. In *Slc52a3*−/− newborn pups immediately after birth, plasma riboflavin concentrations were dramatically lower than in WT pups. These results indicate that Rfvt3-mediated placental riboflavin transport should play a key role in the maintenance of riboflavin homeostasis in fetuses.

Riboflavin is converted to the active coenzyme forms (FMN and FAD), which are indispensable for various metabolic reactions. The initial step in fatty acid β-oxidation is catalyzed by acyl-CoA dehydrogenase, which requires FAD as a co-factor in the reaction. The present study indicates that disruption of *Slc52a3* induced a disorder in fatty acid metabolism, including abnormal levels of plasma acylcarnitine and urinary glutaric acid. Plasma and tissue levels of FAD in *Slc52a3*−/− pups were lower than in WT pups, indicating that riboflavin deficiency significantly affected FAD homeostasis in these mice. Remarkably, this abnormality of fatty acid metabolism in *Slc52a3*−/− pups was clearly improved by riboflavin supplementation of their dams during pregnancy. These results suggest that disruption of *Slc52a3* induced the deficiency of riboflavin in the fetuses and led to the failure in fatty acid β-oxidation. The relevant features of this phenotype in *Slc52a3*−/− mice were reproduced in a clinical report by Bosch *et al.*^16^. Accordingly, three patients with *SLC52A3* mutation exhibited increased plasma acylcarnitines and urine organic acids, and mimicked an inherited disorder affecting mitochondrial fatty acid β-oxidation: i.e. multiple acyl-CoA dehydrogenase deficiency (MADD). All three patients were found to be deficient in plasma flavin in spite of normal dietary intake, and the biochemical abnormalities were clearly improved by riboflavin administration^16^. Taken together, it is strongly suggested that disruption of *Slc52a3* induced the deficiency in riboflavin and led to the abnormality in fatty acid β-oxidation.

Newborn *Slc52a3*−/− mice exhibited hypoglycemia, although transplacental transport of glucose in *Slc52a3*−/− pups was not changed. In addition, amino acids including the gluconeogenic precursor alanine were not decreased in *Slc52a3*−/− pups. It has been suggested that the hypoglycemia was not due to insufficient glucose supplement from the maternal or insufficient gluconeogenic precursors. Hypoglycemia is one of the major clinical signs of fatty acid oxidation defects both in patients and mouse models^26^. Lack of *mitochondrial trifunctional protein* (*MTP*), which catalyzes mitochondrial long-chain fatty acid β-oxidation, leads to neonatal hypoglycemia and sudden death after birth^27^. In addition, deficiency in very long-chain acyl-CoA dehydrogenase (*VLCAD*) or medium-chain acyl-CoA dehydrogenase (*MCAD*) causes hypoglycemia under fasting conditions^28^,^29^. During periods of high metabolic demand, mitochondrial fatty acid β-oxidation is stimulated to preserve glucose, which is an indispensable energy source for the brain^30^. In *LCAD* knockout mice, peripheral tissues utilizes more glucose to compensate for impaired fatty acid oxidation under fasting condition^31^. Enhanced peripheral glycogenolysis has also been observed in patients with *carnitine palmitoyltransferase II* deficiency or *VLCAD* deficiency during prolonged low-intensity exercise^32^,^33^. In several β-oxidation-deficient mice models, hypoglycemia has also been observed. Thus, it is suggested that *Slc52a3*−/− pups might have increased glucose demand due to impaired fatty acid oxidation, resulting in hypoglycemia. We speculated that, in utero, mother helps *Slc52a3*−/− embryos to obtain nutrients and metabolize fatty acid.

Neonatal death is one of the most striking phenotypes in *Slc52a3*−/− mice. Similarly, several mouse models of defects in mitochondrial β-oxidation of fatty acids have been demonstrated to result in neonatal mortality^26^. Disruption of the *MTP* gene results in fatty acid metabolism abnormality and death 6–36 hr after birth^27^. In addition, *MCAD* knockout mice also manifest significant neonatal mortality^29^. Although the precise mechanisms of neonatal loss in these mice remain undetermined, impaired fatty acid oxidation might be lethal in newborn pups.

Recent clinical reports have identified *SLC52A3* mutations in patients with BVVLS^14^,^15^,^16^,^17^,^18^,^19^,^20^,^21^. Although the pathophysiology of BVVLS is not well known, bulbar palsy, sudden hearing loss, facial weakness, respiratory compromise and muscle weakness have been frequently observed in these patients^22^,^34^. Because of early death in *Slc52a3*−/− mouse pups, the several symptoms of BVVLS including hearing loss and muscle weakness could not be clearly evaluated in this study. However, it has also been reported that some patients die in infancy or youth^22^,^34^, and fatty acid metabolism disorders have been observed in some patients^14^,^16^. The neurological symptoms and increased levels of plasma fatty acids have been improved by the administration of a high dose of riboflavin in some cases^14^,^16^,^18^. Thus, the phenotypes of *Slc52a3*−/− mice were similar to those in the patients. While there is no information on the status of BVVLS patients immediately after birth, we assume that some patients with *SLC52A3* mutations might have died from some unknown cause. Although further studies are needed, *Slc52a3*−/− mice could serve as an experimental BVVLS model, and may help clarify its underlying mechanism and lead to development of new drugs for treatment of this disease.

In conclusion, Rfvt3 is involved in placental riboflavin transport and regulates riboflavin homeostasis in neonatal pups in mice. Disruption of *Slc52a3* caused neonatal lethality with hyperlipidemia and hypoglycemia attributable to riboflavin deficiency. These data support that riboflavin supplementation can prevent neonatal death in BVVLS patients with *SLC52A3* mutations, and may help to clarify the mechanisms involved in their neurological symptoms.

## Methods

### Generation of Slc52a3 knockout mice

Animal experiments were conducted in accordance with the *Guidelines for Animal Experiments of Kyoto University*. All protocols were approved by the Animal Research Committee, Graduate School of Medicine, Kyoto University. *Slc52a3*−/− embryos (C57BL/6N-2310046K01Rik^tm1a(KOMP)Wtsi^) were obtained from Knockout Mouse Project repository (*2310046K01Rik*; synonym for *Slc52a3*)^35^. The targeting vector is described in Supplementary Fig. 1a. To confirm the homologous recombination, mouse genomic DNA was isolated from tail biopsies of offspring (F1) using DNeasy Blood and Tissue Kits (Qiagen), and used for the long-range PCR. Long-range PCR analysis was performed using TaKaRa Ex Taq® Hot Start Version (TAKARA BIO) according to the following profile immediately after an initial 3-min denaturation step at 94 °C: 94 °C for 30 sec, 55 °C for 30 sec, 72 °C for 9 min, 32 cycles. The primer sets were as follows: forward primer 5′-GAGTGGCACTTGACATTCGAGGATAAGC-3′ and reverse primer 5′-CACAACGGGTTCTTCTGTTAGTCC-3′ for the 5′ homology arm, and forward primer 5′-TCTATAGTCGCAGTAGGCGG-3′ and reverse primer 5′-GCGGCTTTTACCAAGAATATCAAG-3′ for the 3′ homology arm based on the sequence. Then, F1 *Slc52a3* heterozygous knockout (*Slc52a3*+/−) animals were intercrossed to generate WT, *Slc52a3*+/− and *Slc52a3*−/− mice. To determine the genotype, total DNA was isolated as described above and PCR analysis was performed with a forward primer 5′-TCCTGAGGCAAAAGTGGAAA-3′ and a reverse primer 5′-GGCTGAAATAGCCCACCTTT-3′ for detecting wild-type alleles, and a forward primer 5′-GAGATGGCGCAACGCAATTAAT-3′ and a reverse primer 5′-GGCTGAAATAGCCCACCTTT-3′ for detecting mutant alleles. PCR conditions were as follows: 94 °C for 30 sec, 55 °C for 30 sec, 72 °C for 3 min, 32 cycles. The mice were housed in a temperature-controlled environment with an alternating 12-hr light/dark cycle, and were given a standard chow diet (F-2; Funabashi Farm) and water at libitum before experiments.

### Real-time PCR

Total RNA was isolated from the kidney and placenta using RNeasy Mini Kit (Qiagen), and then was reverse-transcribed. To determine the mRNA expression level of *Slc52a3*, real-time PCR was performed as described previously^8^. TaqMan Gene Expression assays were purchased from Life Technologies: *Slc52a3,* Mm00510189_m1.

### *In situ* hybridization

*In situ* hybridization was performed by the method of Genostaff, Inc. as previously described^13^. Hybridization was performed with *Slc52a3*-specific probes (sequence position; 1045–1645 in the open reading frame) at concentrations of 300 ng/ml in the Prove Diluent-1 (Genostaff) at 60 °C for 16 hr. The sections were counterstained with Kernechtrot stain solution (Muto Pure Chemicals).

### Placental transport of [^3^H]riboflavin in pregnant Slc52a3+/− mice

Transport activity for [^3^H]riboflavin across the placenta of pregnant *Slc52a3*+/− mice, which were mated with *Slc52a3*+/− male mice, at gestation date 16.5 was determined as previously reported^36^. Pregnant dams were anesthetized with an intraperitoneal administration of sodium pentobarbital. Surgery was performed on each animal lying on a heating pad to maintain constant body temperature. Then, radioisotope-labeled riboflavin (500 nM of [^3^H]riboflavin, 0.903 TBq/mmol, Moravek Biochemicals) or glucose (40 μM of D-[U-^14^C]glucose, 11.5 GBq/mmol, GE Healthcare) was administrated as a bolus via the femoral vein (10 mL/kg). Five minutes after administration, fetuses and placentas were removed and weighted. Fetal tails were collected, and used for genotyping as described above. Fetuses were homogenized in saline, and the samples were placed into Ultima Gold scintillation cocktail (PerkinElmer) followed by solubilization in SOLVABLE (Packard Instrument Co.). The radioactivity was measured by liquid scintillation counting, and the levels of [^3^H]riboflavin and D-[U-^14^C]glucose in each fetus were determined and their transporter activities were calculated.

### Riboflavin supplementation in Slc52a3−/− mice

Male and female mice, of both whose genotypes were *Slc52a3*+/−, were intercrossed overnight, and then the successful copulation was verified by observation of a vaginal plug. *Slc52a3*+/− dams were supplemented with 50 mg/L of riboflavin in drinking water *ad libitum* from gestational day 0 to the day of parturition, and the P0 pups were used for the studies. P0 pups were collected immediately after birth or 5–9 hr after birth. The pups were sacrificed by decapitation, and the blood and tissues were collected. For the survival analysis, *Slc52a3*+/− dams were supplemented as described above from gestational day 0 to 3 weeks just before delivery. After birth, the newborn pups were also administered daily with 0.75 mg/kg riboflavin subcutaneously up to P21. Blood was collected from weaned mice by tail snip.

### Measurement of glutaric acid, riboflavin, FMN, FAD, acylcarnitines, glucose and lactate

Blood and urine samples were collected from P0 pups. The glutaric acid levels in urine samples were determined using a gas chromatography mass spectrometer (GC/MS) at Shimadzu Techno-Research Inc. The concentrations of riboflavin, FMN, and FAD in plasma and tissue samples were measured by HPLC as described previously^37^. Plasma acylcarnitine levels were measured by Electrospray tandem mass spectrometry (ESI-MS/MS) at Shimadzu Techno-Research Inc.^38^ with some modifications. Briefly, a total volume of 10 μL methanol/acetone/water (7:7:2) was added to 2.5 μL plasma, and evaporated to dryness for 20 min at 50 °C after standing for 10 min at room temperature. The dried sample was redissolved in an aliquot of 100 μL internal standard solution (LABELED CARNITINE STANDARDS SET B, Cambridge Isotope Laboratories, Inc.). The sample was agitated vigorously for 1 hr and centrifuged. The supernatant was evaporated for 1 hr at 50 °C, and dissolved in 100 μL mobile phase (water/methanol/acetonitrile (20:40:40) containing 0.05% formic acid) for ESI-MS/MS analysis, using LCMS-8040 triple quadrupole mass spectrometer equipped with two LC-30AD HPLC pumps and SIL-30AC autosampler (Shimadzu). Blood glucose and lactate levels were determined using the NIPRO FreeStyle Blood Glucose Monitoring System (NIPRO) and Lactate Pro LT-1710 (Arkray Inc.), respectively.

### Measurement of metabolites in the liver

Liver samples were removed from the P0 pups. Frozen tissue was plunged into 50% acetonitrile solution containing internal standards (H3304-1002, Human Metabolome Technologies) at 0 °C in order to inactivate the enzymes. The tissue was then homogenized and centrifuged (2300 × g, 5 min). Subsequently, the upper aqueous layer was centrifugally filtered through a Millipore 5-kDa cutoff filter to remove proteins. The filtrate was centrifugally concentrated and resuspended in water for capillary electrophoresis coupled to mass spectrometry (CE-MS) analysis. Cationic compounds were measured in the positive mode of CE-TOFMS, and anionic compounds were measured in the positive and negative modes of CE-MS/MS as previously reported^39^,^40^,^41^. Metabolome measurements and hierarchical cluster analysis were performed at a service facility of Human Metabolome Technologies, Inc.

### Statistical analysis

Data are expressed as means ± SD, and differences were statistically analyzed by the unpaired Student’s t-test. Multiple comparisons were performed by one-way ANOVA with either the Bonferroni test (parametric) or Kruskal-Wallis with the Dunn’s test (nonparametric) using GraphPad Prism 5.0 (GraphPad Software Inc.). For Mendelian randomization analysis, the chi-square test with 2-degree freedom was used. For the survival analysis, the differences in survival rates were examined with the log-rank test. Differences where *P* < 0.05 were considered as statistically significant.

## Additional Information

**How to cite this article**: Yoshimatsu, H. *et al.* Disruption of *Slc52a3* gene causes neonatal lethality with riboflavin deficiency in mice. *Sci. Rep.* **6**, 27557; doi: 10.1038/srep27557 (2016).

## Supplementary Material

Supplementary Information

## Figures and Tables

**Table 1:** Fetal and placental wet weights of WT, *Slc52a3*+/−, and *Slc52a3*−/− fetuses at E16.5.

	WT	*Slc52a3*−/−
Fetal weight (mg)	722 ± 22	594 ± 22**
Placental weight (mg)	114 ± 5	102 ± 2*
Fetal to placental weight ratio	6.37 ± 0.26	5.82 ± 0.22

All data are expressed as the mean ± SD.

Values where *P < 0.05 or **P < 0.01 indicate significant difference from WT.

**Table 2:** Plasma acylcarnitine levels in WT and *Slc52a3*−/− mice with (+) and without (−) riboflavin supplementation (RS).

	WT		*Slc52a3*−/−	
	RS (−)	RS (+)	RS (−)	RS (+)
C0	48.9 ± 14.6	47.9 ± 3.3	55.5 ± 14.1	43.8 ± 7.2
C2	7.70 ± 3.35	8.63 ± 1.17	5.67 ± 1.18	4.14 ± 2.96
C3	0.16 ± 1.28	0.11 ± 0.24	0.25 ± 0.39	0.15 ± 0.21
C4	0.20 ± 0.34	1.25 ± 0.99	14.73 ± 7.54^***^	3.88 ± 4.89^††^
C5	0.09 ± 0.09	0.20 ± 0.11	2.81 ± 1.23^***^	0.31 ± 0.29^†††^
C8	0.04 ± 0.07	0.03 ± 0.07	0.14 ± 0.21	0.06 ± 0.09
C14	0.05 ± 0.07	0.03 ± 0.07	1.87 ± 0.69^***^	0.66 ± 0.61^†††^
C16	0.13 ± 0.15	0.05 ± 0.12	0.57 ± 0.74	0.31 ± 0.41

(unit: μM).

In the riboflavin-supplemented group, the pregnant dams were given 50 mg/L of riboflavin in drinking water from gestational day 0. Postnatal day 0 (P0) pups were collected after 5–9 hr of birth.

All data are expressed as the mean ± SD.

Values where ^***^P < 0.001 indicate significant difference from WT (RS (−)).

Values where ^††^*P* < 0.01, ^†††^*P* < 0.001 indicate significant difference from *Slc52a3*−/− (RS (−)).
