# Supplementary material for: Disruption of Slc52a3 gene causes neonatal lethality with riboflavin deficiency in mice
Source: Sci Rep. 2016 Jun 8;6:27557. doi: 10.1038/srep27557 (PMC4897618; doi:10.1038/srep27557)
Supplement: Supplementary Information [file srep27557-s1.pdf]

Disruption of *Slc52a3* gene causes neonatal lethality with riboflavin deficiency in mice

Hiroki Yoshimatsu[1], Atsushi Yonezawa[1*], Kaori Yamanishi[1], Yoshiaki Yao[1], Kumiko Sugano[1], Shunsaku Nakagawa[1], Satoshi Imai[1], Tomohiro Omura[1], Takayuki Nakagawa[1], Ikuko Yano[1], Satohiro Masuda[1,†], Ken-ichi Inui[1,††], Kazuo Matsubara[1]

[1]Department of Clinical Pharmacology and Therapeutics, Kyoto University Hospital, Sakyo-ku, Kyoto 606-8507, Japan

Present affiliation: [†]Department of Pharmacy, Kyushu University Hospital, Fukuoka, 812-8582, Japan, [††]Kyoto Pharmaceutical University, Yamashina-ku, Kyoto 607-8414, Japan

Supplementary Table S1

Plasma and tissue concentrations of riboflavin, FMN, and FAD in *Slc52a3*-/- mice with (+) or without (-) riboflavin supplementation (RS).

In the riboflavin-supplemented group, the pregnant dams were given 50 mg/L of riboflavin in drinking water from gestational day 0. The P0 *Slc52a3*-/- pups were sacrificed 5-9 hr after birth, and plasma and tissue concentrations of riboflavin, FMN and FAD were measured by HPLC. All data are expressed as the mean ± SD.

Values where $*P < 0.05$, $**P < 0.01$, significantly different from RS (-).

	**Riboflavin**		**FMN**		**FAD**	
	**RS (-)**	**RS (+)**	**RS (-)**	**RS (+)**	**RS (-)**	**RS (+)**
**Plasma**	5.38 ± 2.79	80.59 ± 68.41*	81.10 ± 71.69	47.91 ± 38.81	84.52 ± 21.46	91.97 ± 15.94
**Brain**	0.29 ± 0.05	0.85 ± 0.86	0.09 ± 0.02	0.13 ± 0.04*	0.84 ± 0.11	1.17 ± 0.18**
**Lung**	0.77 ± 0.31	1.59 ± 0.62*	0.11 ± 0.10	0.09 ± 0.02	1.61± 0.11	2.16 ± 0.35**
**Heart**	2.46 ± 1.30	4.82 ± 2.00*	0.20 ± 0.13	0.34 ± 0.09*	3.11 ± 0.83	8.70 ± 5.20*
**Liver**	2.48 ± 0.89	4.18 ± 2.04	0.27 ± 0.11	0.33 ± 0.15	4.56 ± 0.49	7.24 ± 2.60*
**Kidney**	1.74 ± 0.42	3.67 ± 1.08**	0.26 ± 0.09	0.40 ± 0.07**	1.74 ± 0.42	2.66 ± 0.61**

(Unit: plasma, µM; tissues, nmol/g tissue)

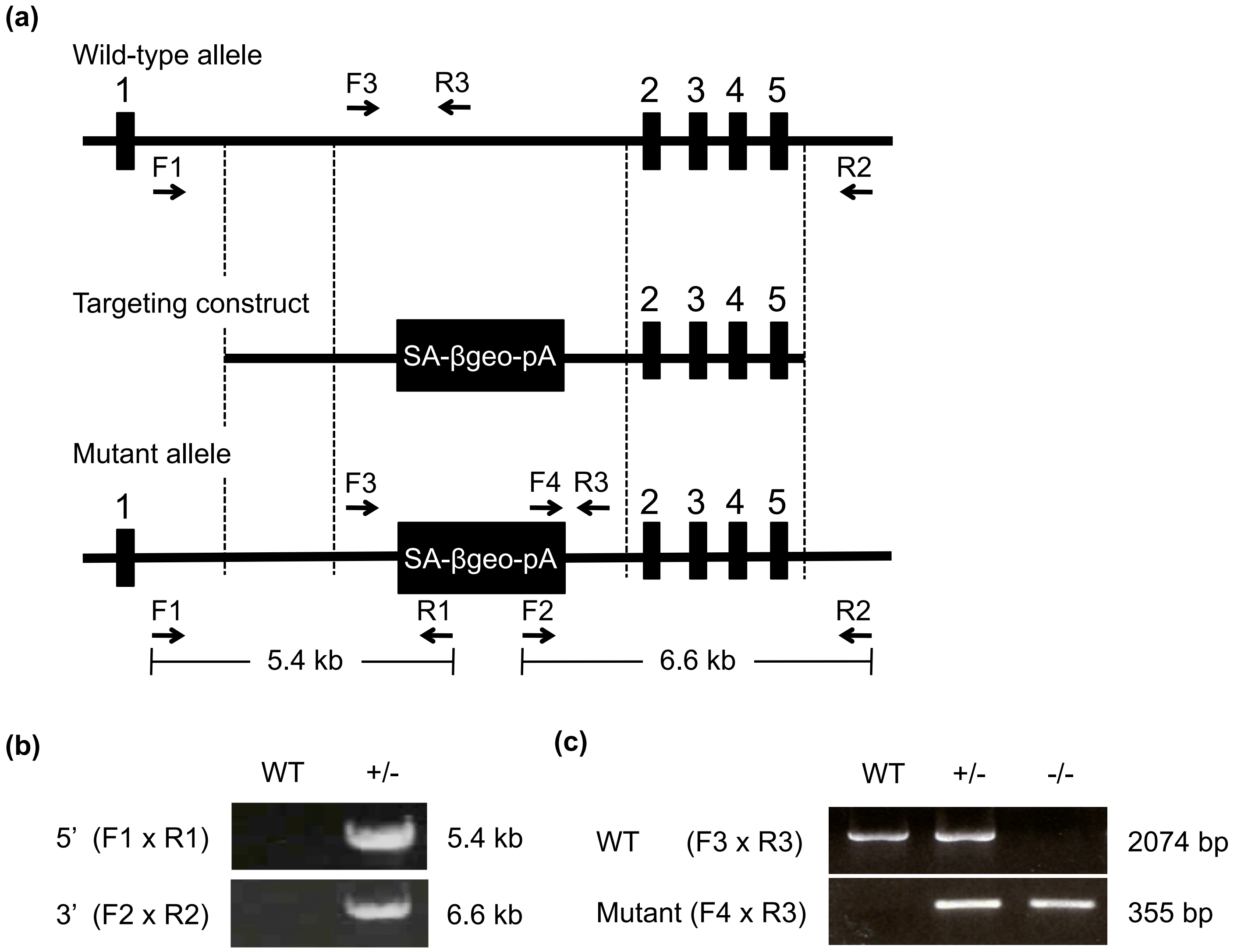

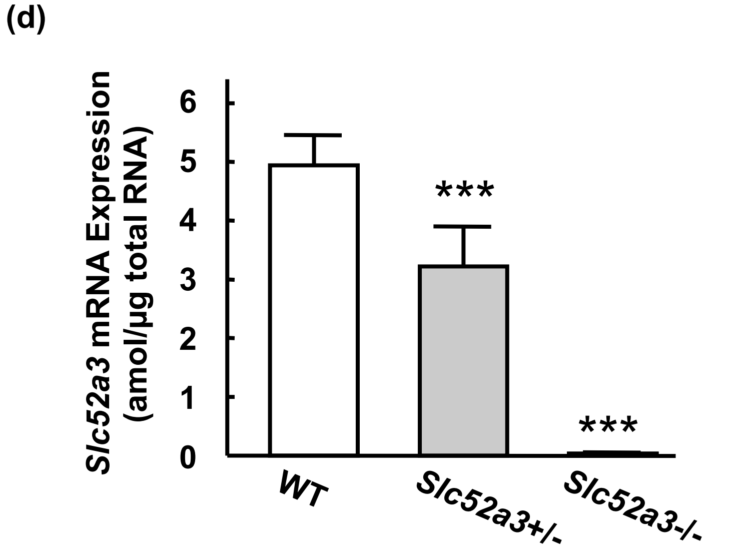

Supplementary Figure S1.
Target disruption of *Slc52a3*. (a) Diagrams of *Slc52a3*-/- construct. Exons are indicated by closed boxes. Primers used for long-range PCR are depicted as arrowheads. (b) Long-range PCR analysis of genomic DNA from mouse tail biopsy samples for confirmation of homologous recombination. (c) Genotyping by PCR analysis. (d) mRNA expression of *Slc52a3* in wild-type (WT), *Slc52a3*+/- and *Slc52a3*-/-. Total RNA isolated from the kidney obtained from newborn pups was reverse-transcribed and mRNA level of *Slc52a3* was determined by real-time PCR. Each column represents the mean ± SD. Values where $***P < 0.001$ indicate significant difference from WT.

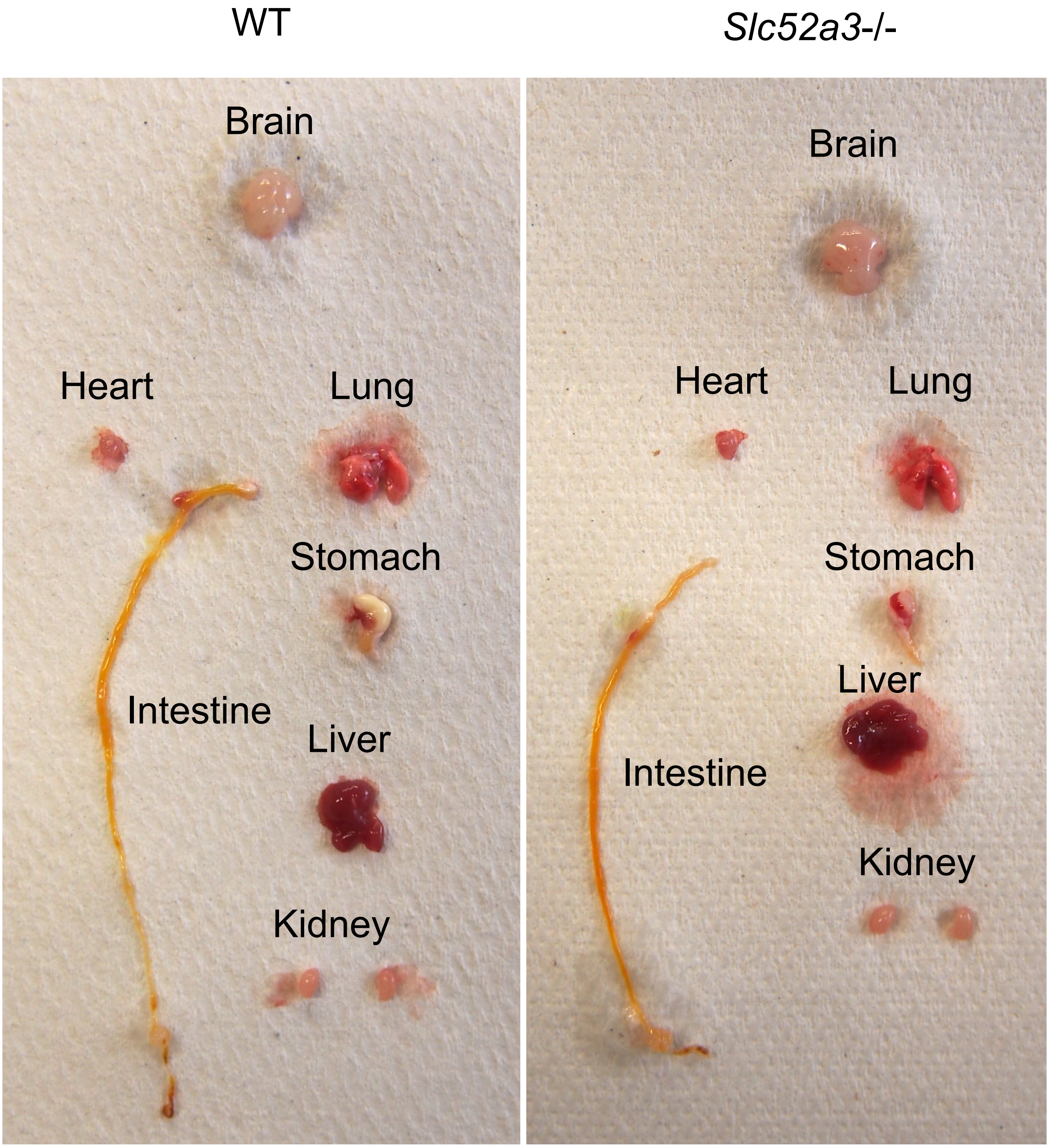

Supplementary Figure S2. Gross appearance of tissues of *Slc52a3*-/- neonatal mice.
Tissues were obtained from WT and *Slc52a3*-/- newborn pups at postnatal day 0.

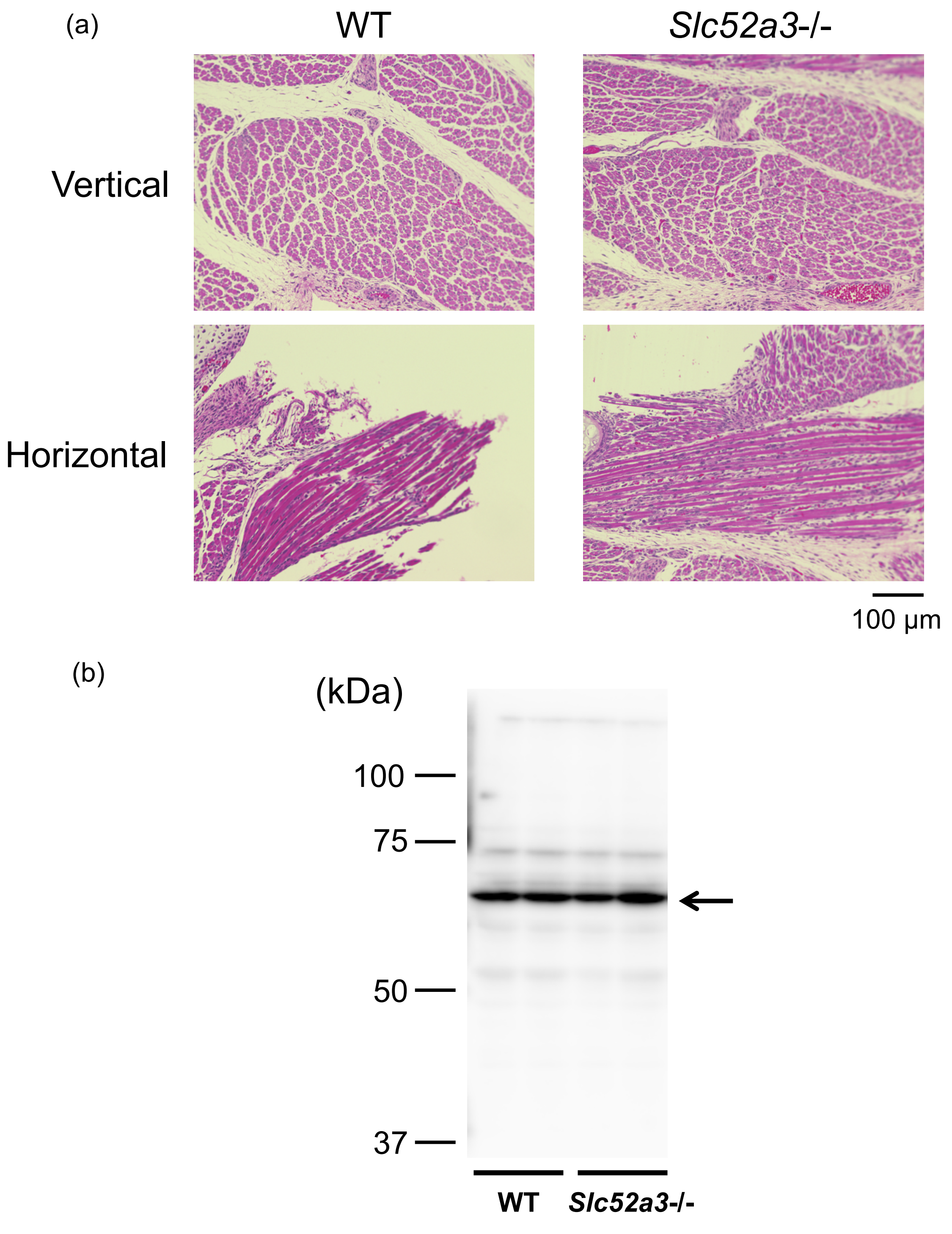

Supplementary Figure S3. Analysis of muscle tissues in *Slc52a3*-/- neonatal mice.
(a) Tissues were collected from WT and *Slc52a3*-/- littermates at P0 were fixed in 10% formalin and stained with hematoxylin-eosin by KAC Co., Ltd. (Kyoto, Japan). Femoral muscles were vertically and horizontally observed. (b) Western blotting analysis of femoral muscles was carried out using anti-apoptosis-inducing factor, mitochondrion-associated 1 (AIFM1) antibody. Femoral muscles were homogenized in RIPA lysis buffer, and equal amouts of protein lysate (30 μg per lane) were analyzed. Primary and secondary antibodies were anti-AIFM1 antibody (Proteintech Group Inc, 17984-1-AP) and horseradish peroxidase-conjugated anti-rabbit IgG (GE Healthcare Bio-Sciences), respectively. Representative photographs are shown.

(a)

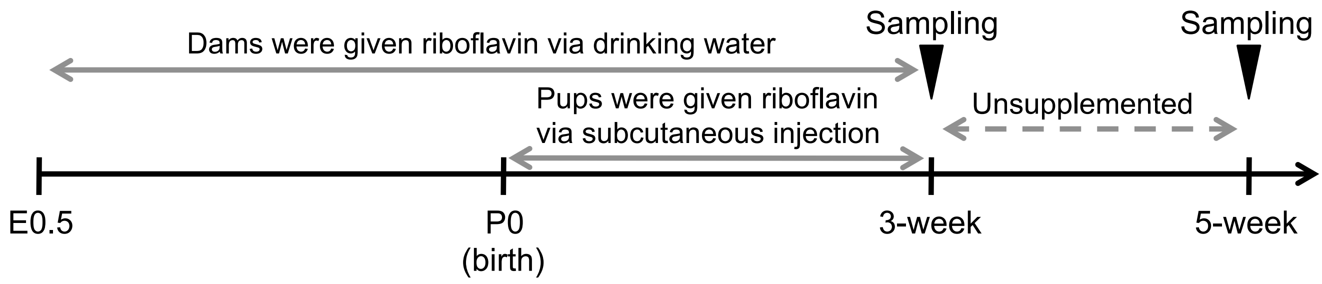

(b)

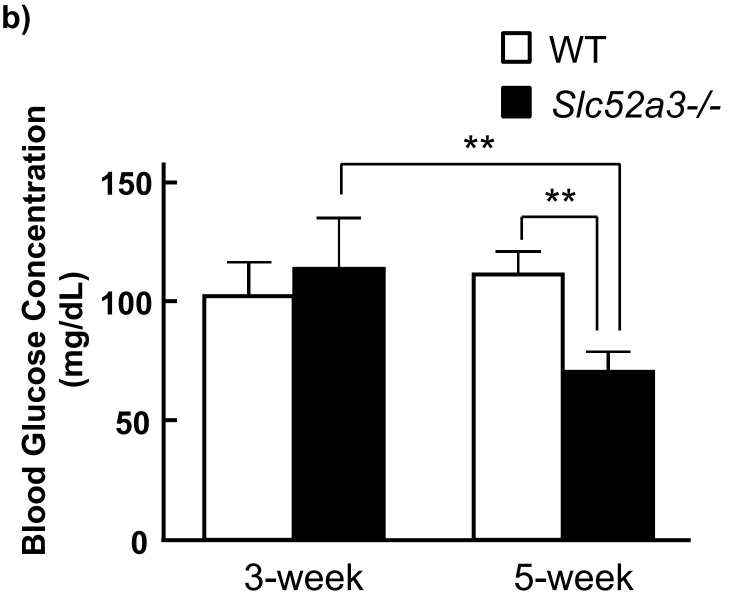

Supplementary Figure S4. Influence of riboflavin supplementation on blood glucose level in *Slc52a3*-/- mice.

(a) Protocol for the animal experiments. *Slc52a3*+/- mice were mated, and pregnant dams were supplemented with 50 mg/L of riboflavin in drinking water *ad libitum* from gestational day 0 to 3 weeks postpartum. Newborn pups were also administered 0.75 mg/kg riboflavin subcutaneously once a day until weaning (3-week). From 3 to 5 weeks after birth, the mice received no riboflavin supplementation. (b) Blood glucose level in WT and *Slc52a3*-/- mice at 3 weeks old (riboflavin-treated condition) or 5 weeks old (riboflavin-untreated condition). Each column represents the mean ± SD (3-week-old WT, n=5; 3-week-old *Slc52a3*-/-, n=4 5-week-old WT, n=5; 5-week-old *Slc52a3*-/-, n=5). Values where $^{**}P < 0.01$ indicate significant difference from controls (WT).
